# The need for open access MRI in psychosis: introducing a new global imaging resource (PsyShareD)

**DOI:** 10.1038/s41537-024-00501-0

**Published:** 2024-10-01

**Authors:** Simon L. Evans, Paul Allen

**Affiliations:** 1 School of Psychology, Faculty of Health and Medical Sciences, University of Surrey, Guildford, UK; 2 Dept of Neuroimaging, Institute of Psychiatry, Psychology and Neuroscience, King’s College London, London, UK

**Keywords:** Biomarkers, Psychosis

Link to video interview: https://vimeo.com/1006730474/069d14a680?share=copy.

Schizophrenia and other psychotic disorders are highly debilitating and while current treatments can often ameliorate the positive symptoms such as hallucination and delusions, many of those affected cannot function well in society - and this has a devastating impact on a persons’ well-being. It also means that the societal and economic costs associated with these conditions are disproportionately large. Poor functional recovery and limited treatment efficacy reflect a general lack of aetiological understanding.

Despite decades of research investment and a large number of Magnetic Resonance Imaging (MRI) studies having been conducted, the brain morphological abnormalities which underpin psychosis, and how these link to symptoms and impairments in cognitive domains, remain poorly characterised and understood. Inconsistent and conflicting findings abound in the neuroimaging literature. This lack of clarity means that we still cannot say with confidence which are the neuroanatomical markers and mechanisms that underlie psychosis risk and symptomology. Knowledge of how these evolve across the disease course, and the links between brain structural properties and specific symptoms is essential, if we are to develop the aetiological and mechanistic understanding necessary for advancing early detection and treatment approaches.

The heterogenous nature of schizophrenia and related psychotic disorders, compounded by small sample sizes and methodological inconsistencies, contribute to the lack of parsimony in findings. To try and address this, traditional meta-analyses pool statistics across neuroimaging studies, but as effect sizes are calculated based only on whatever summary statistics are available from each study, the power to test specific hypotheses is restricted due to heterogeneity in data analysis methods and the regions of interest included. This is particularly important in key regions such as hippocampus and prefrontal cortex, given the many functionally distinct sub-regions that make up those structures. This lack of precision means that meta-analyses have also been ineffective at delineating the specific brain structural changes that occur with disease progression.

In response to this, multi-centre collaborations (such as ENIGMA, PSYSCAN, and others) have emerged recently, to provide more robust insights based on pooled analyses of individual subject data. To address the shortcomings of traditional meta-analyses, these initiatives usually employ a standardised analysis pipeline on the data from each site, thus improving analytic power and consistency. For all these initiatives, maximising the N, and the inclusion of multiple sites, is a priority and major strength, meaning that analyses are usually better-powered than the vast majority of single studies, and that results are more representative of the global patient population and therefore more generalisable. These initiatives vary in terms of global reach; while PSYSCAN data derives mostly from Europe and Australia^1^, ENIGMA has better global reach, although the N and diversity of sites vary across studies. The larger analyses from the ENIGMA-schizophrenia working group include collaborators from South Africa, South Korea and Singapore^2^, Japan and China^3^, and Brazil^4^. This latter study, in nearly 4500 individuals with schizophrenia across 39 study samples, provides an excellent example of how multisite collaborative efforts can leverage the resultant statistical power to generate robust insights, in this case around regional cortical thickness differences in schizophrenia and their associations with medication dose, symptom severity, and duration of illness - thus clarifying a hitherto heterogenous and inconsistent literature. Likewise, the ENIGMA clinical high-risk (CHR) working group has provided valuable insights into the neuroanatomical indicators of psychosis risk, based on data from 1792 CHR individuals across 31 sites; while the majority were from Europe/USA, Russia, Japan, Singapore, Columbia and Mexico were also represented^5^.

Multicentre initiatives have thus made some excellent preliminary progress in advancing knowledge around the neuroanatomical underpinnings of psychosis. However, generating papers from these can be difficult and time-consuming, since datasets either have to be transferred and then analysed, or analysed separately by each site before results can be interpreted and published. Crucially, the issue is that these initiatives are not truly open-access, since data are not stored centrally and made widely accessible; although supporting data are sometimes made available alongside publications, most initiatives do not usually afford non-consortia members the ability to conduct analyses on their data. This makes it difficult for aspiring researchers who are not based at major research centres to conduct well-powered analyses. This problem is compounded by the fact that there is a major lack of open-access neuroimaging repositories in psychosis: those that exist (e.g. http://schizconnect.org/) are severely limited in terms of size, global reach, and what data and support is available to researchers. Given the large volumes of relevant ‘legacy’ neuroimaging data that exists globally, this really holds back the field, both in terms of capacity building for early-career researchers and for leveraging these data to drive reliable hypothesis testing and knowledge generation. To make progress, enhance research capacity, and foster new collaborations, there is a need to ensure open and equitable access to a well-resourced MRI depository covering a diverse range of schizophrenia and psychosis populations.

Recognising this, we present an ambitious new data-sharing initiative: the **Psy**chosis MRI **Share**d **D**ata Resource (PsyShareD), which is currently combining a large number of high-quality, pre-existing MRI datasets, with linked demographic, clinical and cognitive data, into one free-to-access resource. PsyShareD is currently funded by a Partnership Grant from the UK’s Medical Research Council (MR/X010651/1) and aims to address the issues highlighted above.

As an open-access resource, PsyShareD data will be accessible to researchers worldwide following a straightforward application process. Also, for most datasets, contributing centres provide raw structural imaging data, rather than individual-level summary statistics based on their own processing. This allows a more rigorous harmonisation of data, followed by a unified processing and analysis pipeline. A major focus of PsyShareD is global reach: to address the bias towards Western cohorts, PsyShareD actively seeks contributors from under-represented populations. In addition, PsyShareD is broad in scope: it includes people with schizophrenia (SCZ), first episode psychosis (FEP), bipolar disorder (BPD), clinical high risk (CHR), schizotypy (SZT), the experience of childhood trauma (CT), and healthy control (HC) populations, thus allowing a wide range of analyses across illness and risk stages. Longitudinal data is particularly valuable and included where available.

PsyShareD uses a secure transfer and storage infrastructure. Collaborators (Partners) provide anonymised T1 data which is then quality-checked by the PsyShareD team, and processed using FreeSurfer 7.3.2 (https://surfer.nmr.mgh.harvard.edu/), feature-level data (including cortical and sub-cortical volumes, cortical thickness, and surface area data) is extracted and harmonised using ComBat software (ComBatHarmonization). Image-level harmonisation (using CycleGAN, https://junyanz.github.io/CycleGAN/, and HACA3^6^) is also applied to the T1 data to broaden the range of analyses possible (e.g. voxel-based morphometry). Linked demographic and clinical data is available for all datasets. Cognitive/IQ data and other measures (e.g. functional capacity) are also provided where available; comprehensive and searchable catalogues show what linked data is available for each dataset, via the website (https://psyshared.com/). At the time of writing, PsyShareD has already received data from 9 sites, more will be added over the coming months and we anticipate that PsyShareD will host around 3500 T1 scans by the time it is launched in late 2024.

PsyShareD continues to actively seek further site Partners to contribute their valuable data. We encourage researchers who hold MRI datasets in any of the populations described above to please contact us; we will facilitate secure data transfer. Partners will benefit in terms of authorships, as we stipulate that all resultant publications include contributing site Partners as co-authors. Partners will have the opportunity to review (and, if interested, contribute to) draft publications that use their data before the Psy-ShareD team approves submission.

Our aim is for PsyShareD to be a vital (and growing) resource for the psychosis research community over the long term, potentially also expanding to include other imaging modalities and populations. Also, maximising accessibility is a priority. To facilitate the usage of Psy-ShareD data, we will be providing support and training resources for potential users, allowing less experienced researchers and doctoral students to use the resource and conduct analyses. A workshop is planned for November 2024. If you are interested in becoming a site Partner, or accessing the resource, please visit the website: (https://psyshared.com/) for more information and updates.

## References

[1] Slot, M. I. E. et al. A naturalistic cohort study of first-episode schizophrenia spectrum disorder: A description of the early phase of illness in the PSYSCAN cohort. *Schizophr. Res* **266**, 237–248 (2024). 38431986 10.1016/j.schres.2024.02.018

[2] Constantinides, C. et al. Brain ageing in schizophrenia: evidence from 26 international cohorts via the ENIGMA Schizophrenia consortium. *Mol. Psychiatry* **28**, 1201–1209 (2023). 36494461 10.1038/s41380-022-01897-w PMC10005935

[3] Kelly, S. et al. Widespread white matter microstructural differences in schizophrenia across 4322 individuals: results from the ENIGMA Schizophrenia DTI Working Group. *Mol. Psychiatry* **23**, 1261–1269 (2018). 29038599 10.1038/mp.2017.170 PMC5984078

[4] van Erp, T. G. M. et al. Cortical brain abnormalities in 4474 individuals with schizophrenia and 5098 control subjects via the enhancing neuro imaging genetics through Meta Analysis (ENIGMA) Consortium. *Biol. Psychiatry* **84**, 644–654 (2018). 29960671 10.1016/j.biopsych.2018.04.023 PMC6177304

[5] Group, E. C. H. R. f. P. W. et al. Association of structural magnetic resonance imaging measures with psychosis onset in individuals at clinical high risk for developing psychosis: An ENIGMA Working Group Mega-analysis. *JAMA Psychiatry* **78**, 753–766 (2021). 33950164 10.1001/jamapsychiatry.2021.0638 PMC8100913

[6] Zuo, L. et al. HACA3: A unified approach for multi-site MR image harmonization. *Comput. Med. Imaging Graph* **109**, 102285 (2023). 37657151 10.1016/j.compmedimag.2023.102285 PMC10592042

